# Genes regulated by estrogen in breast tumor cells *in vitro *are similarly regulated *in vivo *in tumor xenografts and human breast tumors

**DOI:** 10.1186/gb-2006-7-4-r28

**Published:** 2006-04-07

**Authors:** Chad J Creighton, Kevin E Cordero, Jose M Larios, Rebecca S Miller, Michael D Johnson, Arul M Chinnaiyan, Marc E Lippman, James M Rae

**Affiliations:** 1 Bioinformatics Program, University of Michigan Medical Center, Ann Arbor, MI 48109, USA; 2 Division of Hematology Oncology, Department of Internal Medicine, University of Michigan Medical Center, Ann Arbor, MI 48109, USA; 3 Department of Oncology, Georgetown University, Washington, DC 20007, USA; 4 Department of Pathology, University of Michigan Medical Center, Ann Arbor, MI 48109, USA

## Abstract

**Background:**

Estrogen plays a central role in breast cancer pathogenesis. Although many studies have characterized the estrogen regulation of genes using *in vitro *cell culture models by global mRNA expression profiling, it is not clear whether these genes are similarly regulated *in vivo *or how they might be coordinately expressed in primary human tumors.

**Results:**

We generated DNA microarray-based gene expression profiles from three estrogen receptor α (ERα)-positive breast cancer cell lines stimulated by 17β-estradiol (E2) *in vitro *over a time course, as well as from MCF-7 cells grown as xenografts in ovariectomized athymic nude mice with E2 supplementation and after its withdrawal. When the patterns of genes regulated by E2 *in vitro *were compared to those obtained from xenografts, we found a remarkable overlap (over 40%) of genes regulated by E2 in both contexts. These patterns were compared to those obtained from published clinical data sets. We show that, as a group, E2-regulated genes from our preclinical models were co-expressed with ERα in a panel of ERα+ breast tumor mRNA profiles, when corrections were made for patient age, as well as with progesterone receptor. Furthermore, the E2-regulated genes were significantly enriched for transcriptional targets of the *myc *oncogene and were found to be coordinately expressed with Myc in human tumors.

**Conclusion:**

Our results provide significant validation of a widely used *in vitro *model of estrogen signaling as being pathologically relevant to breast cancers *in vivo*.

## Abstract 2

Estrogen-regulated gene expression profiles of *in vivo *breast tumor cell lines and *in vitro *xenografts and breast tumors are remarkably similar.

## Background

Estrogenic hormones are key regulators of growth, differentiation, and function in a wide array of target tissues, including the male and female reproductive tracts, mammary gland, and skeletal and cardiovascular systems. Many of the effects of estrogens are mediated via their nuclear receptors, estrogen receptor (ER)α and ERβ. The estrogen receptors mediate a number of effects within the cell, mainly by altering the transcription of genes via direct interaction with their promoters or through binding to other proteins, which in turn interact with and regulate gene promoters [1]. It has been well-established that estrogen plays a significant role in breast cancer development and progression [2]. Increased lifetime exposure to estrogen is a factor in breast cancer risk [3], and drugs that block the effects of estrogen can inhibit the growth of hormone dependent breast cancers and prevent breast cancer [4].

Although much is known about the role of estrogen signaling in breast cancer proliferation, it is still not known which genes are critical for breast pathogenesis. One goal that would help our understanding of the role of estrogen in breast cancer is to characterize the ERα-mediated transcriptional regulatory network. Several studies have been published using DNA microarrays to identify ERα-regulated genes by monitoring the global mRNA expression patterns in breast cancer cells stimulated by estrogen [5-11]. Beyond cataloging the individual genes in the ERα gene network, much could be discovered by considering the gene expression patterns as a whole and how patterns of estrogen regulation may relate to patterns obtained from mRNA profiling studies of other experimental systems and of human tumors. In particular, we examine here how estrogen-induced mRNA expression patterns observed in *in vitro *cell line models correspond to expression patterns in breast tumors *in vivo*, especially in ERα+ breast tumors. We also show how the transcriptional program of estrogen response *in vitro *is observed in large part in an *in vivo *xenograft experimental model. Furthermore, we show an enrichment of estrogen signaling target genes for genes transcriptionally activated by the *myc *oncogene.

## Results

### The global gene expression profile of estrogen response in multiple breast cell lines shows temporal complexity

We studied the gene expression patterns induced in three separate ERα-positive, estrogen dependent breast cancer cell lines (MCF-7, T47D and BT-474) grown in steroid-depleted medium or in the presence of 17β-estradiol (E2). After treatment for intervals varying from 1 to 24 hours, total RNA was extracted from the cells (MCF-7, 10 different RNA samples in total; T47D, 14 samples in total; BT-474, 10 samples in total) and analyzed using Affymetrix Genechip Arrays representing 22,283 mRNA transcripts (12,768 unique named genes). We sought genes with expression patterns common to all three cell lines that were correlated with the proliferative behavior of the cells in response to E2 treatment. Expression values within each cell line were first transformed to standard deviations from the mean, in order to compensate for cell line-specific, but not E2-specific, differences. As observed elsewhere [7], we anticipated that E2-induced mRNA expression changes would be temporally complex, occurring at various time points, and so as an initial selection for differentially expressed genes, we compared the 4, 8, 12, and 24 hour time points across all three cell lines with the 0 time point of E2 treatment. Genes that showed significant up- or down-regulation (*p *< 0.01) for at least one time point were selected for further analysis; 1,989 transcripts (representing 1,592 unique named genes) showed up-regulation and 1,516 transcripts (1,277 genes) were down-regulated (the complete list is provided in Additional data file 1).

As we tested about 22,000 genes for significance of expression, many genes may give a nominally significant *p *value by chance alone. Permutation of the sample labels indicated that on the order of 25% of the 3,501 transcripts (4 transcripts of the 1,989 that showed up-regulation at one time point also showed down-regulation at another time point) selected by our above criteria might be spurious. Alternatively, we might have used a significance threshold of *p *< 0.001 instead of 0.01, which yielded 1,172 significant transcripts with 9% expected false discovery rate (FDR). Specifying criteria for selecting a statistically significant set of genes is a balance between false negatives and false positives. Our approach was to use the less stringent threshold of *p *< 0.01, yielding fewer false negatives. As described below, we integrated our gene sets with results from other mRNA profile datasets, which could add more significance to a given gene that may have only nominal significance in our initial dataset.

**Figure 1:**
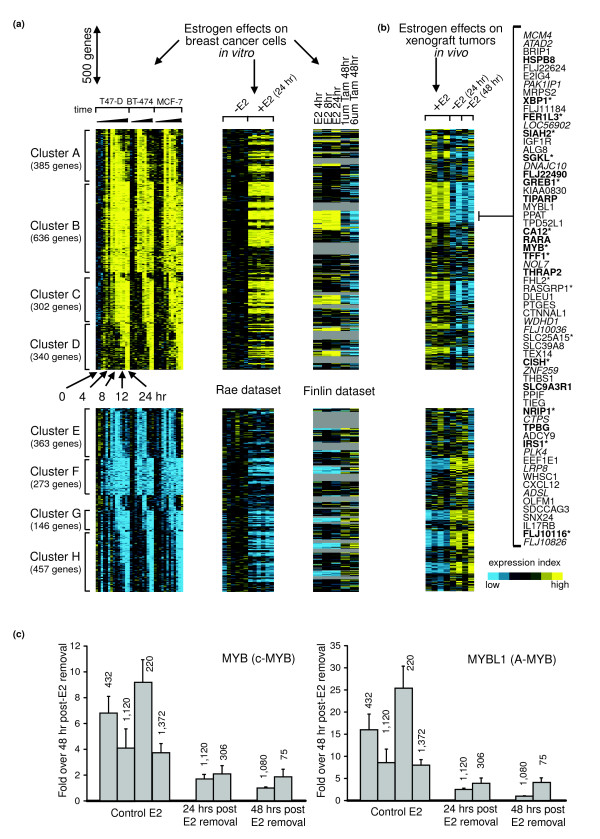
Gene expression signatures of estrogen regulation *in vitro *and *in vivo*. **(a) **Expression data matrix of genes showing either induction or repression by E2 *in vitro *(*p *< 0.01 at 4, 8, 12, or 24 hour time points). Each row represents a gene; each column represents a sample. The level of expression of each gene in each sample is represented using a yellow-blue color scale (yellow, high expression); gray indicates missing data. Shown alongside our *in vitro *time course dataset are the expression values for the corresponding genes in two independent mRNA profile datasets of E2-treated breast cells ('Rae' dataset from [5] of cell lines MCF-7, BT-474, and T47-D; 'Finlin' dataset from [11] of MCF-7). **(b) **Alongside the *in vitro *datasets are the corresponding values for MCF-7 xenografts with or without E2 supplementation (E2 withdrawn for 24 hours and 48 hours in the -E2 group). Genes in cluster 'B' (a) that show significant up-regulation by E2 in each of the four datasets are listed (an asterisk indicates positively correlated (*p *< 0.05) with age-correlation ERα expression (Figure 2); bold type indicates having higher expression in ERα+ compared to ERα- breast tumors (*p *< 0.01) according to the 'van't Veer' dataset from [15]); italics indicates having higher expression in ERα- compared to ERα+ breast tumors). **(c) **Genes *MYB *(c-myb) and *MYBL1 *(A-MYB) are regulated by E2 *in vivo*. Expression patterns for genes from (b) were validated by RT-PCR. Shown are the mean and standard deviation of individual samples assayed in triplicate. Tumor volumes (expressed in mm^3^) are shown above each bar.

We clustered the 3,501 putative E2 transcriptional targets using a supervised method whereby each transcript was assigned to one of the following expression patterns of interest: transcripts induced or repressed early (within 4 hours) but that return to baseline expression before 24 hours (Figure 1a, clusters A and E, respectively); transcripts induced or repressed early (within 4 hours), but with sustained induction or repression through 24 hours (clusters B and F); transcripts induced or repressed through 24 hours beginning at intermediate time points (around 8 hours; clusters C and G); and transcripts induced or repressed beginning at later time points (12 to 24 hours; clusters D and H). Nearly all of the transcripts could be assigned to one of these eight clusters (four clusters for the up-regulated genes, four for the down-regulated genes). One set of genes that did not fall into the above clusters showed up- or down-regulation at only the 8 hour time point (Figure 1a), though these genes were relatively few and no interesting patterns were found for them with respect to other profile datasets examined (described below). The clustering pattern was visualized as a color matrix (Figure 1a), with genes in the rows and experiments in the columns, and with yellow representing high expression and blue representing low expression.

We examined each of the eight E2-regulated gene clusters for significantly enriched (that is, over-represented) Gene Ontology (GO) annotation terms [12] for clues as to the processes that may underlie the coordinate expression of these genes. In the cluster 'B' genes (Figure 1a), representing 636 unique named genes (750 transcripts) induced early with sustained induction by E2, significant GO terms (*q*-value < 0.02) included terms related to ribosomal function and RNA and protein processing, including 'ribosome biogenesis' (14 genes found out of 34 represented in the entire set of profiled genes), 'RNA metabolism' (28/212), and 'protein folding' (19/145), as well as 133 genes with 'nucleic acid binding' function (1,907 total). For the cluster 'E' genes (363 unique, 411 transcripts; repressed at 4 hours but returning to baseline by 24 hours), significant GO terms included 'transcription factor activity' (39/658), 'development' (60/1275), and 'cell adhesion' (27/452). Cluster 'H' genes (repressed at 12 to 24 hours) were enriched for genes located in the 'Golgi apparatus' (30/274). No significant GO terms were found for cluster 'A' genes (induced early but returning to baseline by 24 hours; 385 unique, 435 transcripts), cluster 'F' genes (repressed at 4 hours; 273 unique, 308 transcripts), or cluster 'G' genes (repressed at 8 hours; 146 unique, 157 transcripts).

For the cluster 'C' genes (302 unique, or 346 transcripts) and the cluster 'D' genes (340 unique, or 381 transcripts), which showed sustained induction by E2 at 8 hours and 12 to 24 hours, respectively, significant GO terms found in both clusters included terms related to cell division, including 'cell cycle' (cluster C:53, cluster D:48, 593 total), 'cell proliferation' (C:59, D:65, 879 total), 'mitosis' (C:17, D:16, 98 total), and 'DNA replication' (C:17, D:23, 154 total). An observed enrichment of cell cycle-related genes within the C and D clusters makes intuitive sense, as breast cancer cells stimulated with estrogen will begin to divide and proliferate by 24 hours. Consistent with the GO term search results, when referring to a dataset profiling gene expression during the cell division cycle [13], we found an enrichment for genes showing periodic expression during the cell cycle (618 genes total) within the B, C, and D clusters, with the highest extent of enrichment in C and D (B, 53 genes, *p *= 8E-06; D, 54, *p *= 3E-17; E, 53, *p *= 3E-14).

### Genes regulated by estrogen in breast cancer cell lines *in vitro *are also estrogen-regulated in xenograft tumors *in vivo*

We sought to determine whether the genes showing regulation by estrogen *in vitro *could also be E2-regulated *in vivo*. MCF-7 cells were grown as xenografts in ovariectomized athymic nude mice implanted with sustained-release E2 pellets. After measurable tumors were established (approximately 4 weeks), the mice were randomized into control (continued E2 supplementation, four mice) or E2 withdrawal (surgical removal of pellet, four mice) groups; tumors 24 hours and 48 hours later were collected and profiled for global mRNA expression using Affymetrix arrays (eight profiles in all).

We compared the mRNA profile data from the tumor xenografts (with and without E2) side-by-side with our data for E2-regulated genes *in vitro*. We observed many of the same genes appearing regulated by E2 *in vivo *in the same direction as what we observed *in vitro *(Figure 1b), thereby demonstrating how these two very different experimental models can yield similar results. Of the 435 cluster A transcripts derived from the *in vitro *data (Figure 1a), 22% showed up-regulation by E2 in tumor xenografts; of the 750 cluster B transcripts, 45%; of the 346 cluster C transcripts, 48%; and of the 381 cluster D transcripts, 27%. Similarly, while only 4% of the 411 cluster E transcripts showed down-regulation by E2 *in vitro*, the percentage for the 308 cluster F transcripts was 32%; for the 157 cluster G transcripts, 50%; and for the 499 cluster H transcripts, 42%. We validated our xenograft microarray results using real-time PCR analysis for genes *MYB *(v-myb myeloblastosis viral oncogene homolog (avian), or c-myb) and *MYBL1 *(v-myb homolog-like 1, or A-MYB) (Figure 1c). *GREB1*, another gene arising from our analysis, had been previously validated by our group as being induced by E2 *in vivo *[5].

We compared our xenograft and *in vitro *mRNA profile data with two other independent *in vitro *profile datasets from previous studies: one dataset generated by our group [5] of three ERα-positive cell lines (MCF-7, T47D, and BT-474) grown in steroid-depleted medium or in the presence of E2 for 24 hours (the 'Rae' dataset); and another dataset from a similar experiment carried out by a different group using a different microarray platform (cDNA) [7] and MCF-7 cells treated with E2 or ICI 182,780 (the 'Finlin' dataset). When viewing the qualitative results of our original *in vitro *dataset side-by-side with those of the other three datasets, we found that most of the genes in our E2-regulated gene sets showed E2-regulation in the same direction in at least one other dataset (Figure 1a), thereby adding confidence to these genes as being *bona fide *E2 targets. Of the 750 cluster B transcripts from the original dataset, 73 (63 unique genes) showed E2 induction in each of the other three datasets (xenograft dataset, *p* < 0.05; Rae dataset, *p* < 0.05; Finlin dataset, average fold change >1.4). Many of the cluster B transcripts were significant in one or two of the other three datasets. In particular, we identified 172 cluster B transcripts (148 unique genes) that were significant in the xenograft and Rae datasets but not in the Finlin dataset, 47 of these transcripts not being represented in the Finlin dataset. Of the 750 cluster B transcripts, 215, or 29%, did not show significant regulation in any of the other three datasets. We had anticipated a 25% FDR for our initial gene selection (see above), and so we might expect this set of 215 transcripts to be highly enriched for transcripts giving spurious results due to multiple gene testing.

Some genes appeared regulated by E2 *in vitro *but not *in vivo*; 162 of the cluster B transcripts (142 unique genes) also showed significance in the Rae dataset but not in the xenograft dataset. Similarly, our data might reveal genes that appear regulated by estrogen *in vivo *but not *in vitro*. Of the 459 most significantly E2-induced transcripts in the *in vivo *dataset (*p *< 0.05, fold change >1.5), 97 did not show any similar trend towards significance in either of the *in vitro *Affymetrix datasets (*p *> 0.20). Possible reasons for this disparity have been mentioned above; however, these putative *in vivo*-specific targets of E2 may be enriched for false positives, and so genes of interest in this set would need to be independently validated.

In our analyses below involving human tumor profile data, we focused on our sets of *in vitro *E2-regulated genes, as we wanted to determine whether genes regulated at different time points might show differences with respect to patterns in human tumors. However, we did find the set of genes induced by E2 *in vivo *to generally show the same patterns (results not shown) described below for cluster B (the cluster of early and sustainable induced genes).

### A significant number of genes induced by estrogen *in vitro *are correlated with age-corrected ERα mRNA expression in ERα+ human breast tumors *in vivo*

We next examined the mRNA expression patterns of our *in vitro *E2-regulated gene sets in human breast tumors to determine how these genes might be pathologically relevant (that is, relevant from a disease standpoint). We hypothesize that ERα+ breast cancers would express a significant number of the E2-regulated genes observed in our *in vitro *data set. Since pathologically classified ERα+ breast cancers have been shown to express varying levels of ERα both at the protein and mRNA level, we examined the dataset from van de Vijver *et al*. [14] of 295 patient breast tumor mRNA expression profiles, focusing first on the subset of 226 ERα+ tumor profiles to determine whether E2-regulated genes might be correlated with ERα expression in these tumors. ERα mRNA level had been measured by a 60-mer oligonucleotide on the microarray, which was observed to correlate highly with the measured protein level [15].

**Figure 2:**
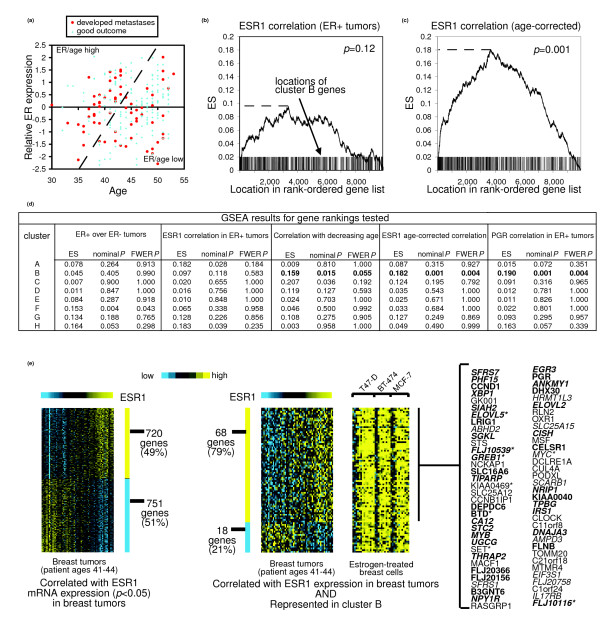
A significant number of genes induced by E2 *in vitro *are correlated with age-corrected mRNA expression of ERα in human breast tumors. **(a) **Scatter plot of ERα expression versus age in ERα+ breast tumor samples from the profile data from [14]. The dotted line is used to stratify the samples into ER/age high (above the line) and ER/age low (below the line) groups. Figure adapted from [18]. **(b) **GSEA of the cluster B genes (induced early by E2 and sustained over time; Figure 1a) against the overall ranking of genes according to similarity with ERα mRNA (*ESR1*) expression in the cohort of ERα+ breast tumor profiles from [14]. The ES statistic (the maximum of the ES running sum) is high if many genes in the set of interest appear near the top of the ranked list. Vertical bars along the ES plot denote occurrences of a cluster B gene. **(c) **GSEA of the cluster B genes against gene ranking by similarity with *ESR1 *expression as corrected for patient age (using dotted line in (a)). **(d) **GSEA results for estrogen-regulated gene clusters A to H against four different gene rankings tested. The FWER *p*-value corrects for multiple gene set testing. **(e) **Enrichment of cluster B genes within the set of genes showing positive correlation (*p *< 0.05) with *ESR1 *expression in a set of tumor profiles from patients within a narrow age range of 41 to 44 years. In the gene list: an asterisk indicates negatively correlated (*p *< 0.05) with age in ERα+ tumors; bold type indicates having higher expression in ERα+ compared to ERα- breast tumors (*p *< 0.01); and italics indicate induced by E2 in breast tumor xenografts (*p *< 0.05; Figure 1b).

Using the breast tumor profile dataset, we constructed a list of the profiled genes ordered according to similarity with ERα mRNA expression (that is, genes having high expression when ERα has high expression and having low expression when ERα has low expression would be at the top of this list). We next used Gene Set Enrichment Analysis (GSEA) [16,17] to capture the position of genes in the E2-induced cluster B genes (induced within 4 hours, Figure 1a) within this ordered list. GSEA determines whether a rank-ordered list of genes for a particular comparison of interest (for example, correlation with ERα in human breast tumors) is enriched in genes derived from an independently generated gene set (for example, the cluster B genes). In fact, we did not see a significant enrichment of cluster B genes within the top tumor ERα correlates, though a trend towards significance was evident (*p *= 0.12, Figure 2b). This result caused us to consider other factors in addition to ERα expression to assess the amount of estrogen signaling in tumors.

Along with ERα status, age is thought to have an important impact on survival in breast cancer, with younger patients having a poorer outcome [18]. We might expect a trend of tumors from younger patients having more estrogen signaling, as younger patients have higher levels of estrogen. In fact, when ranking the genes in the breast tumor dataset by inverse correlation with age at diagnosis (genes at the top of the ranked list would be most highly expressed in younger patients compared to older patients), we did see an enrichment of the E2-induced cluster B genes within genes more highly expressed in young patients (GSEA nominal *p *= 0.015, FWER (Family-Wise Error Rate) *p *= 0.055). Besides cluster B, none of the *in vitro *E2-regulated gene clusters showed similar coordinate expression in either younger or older patients (Figure 2d).

Gene expression data may be combined with other clinical variables to reveal patterns that might not have been observed when considering the variables in isolation. In the study by Dai *et al*. [18], one group used ERα level and its variation with age to subdivide the patients represented in the tumor profile dataset used in this study. When the ERα level obtained from the microarray measurements was plotted versus age for the ERα+ patients, the patients appeared distributed into two distinct subpopulations (Figure 2a). The profiles were stratified into an 'ER/age high' group (meaning high ERα expression for their age), and an 'ER/age low' group, with patients in the 'ER/age high' group having poor overall outcome. Based on these previous findings, we hypothesized that patients in the 'ER/age high' group would have higher expression of E2-induced genes. We developed an 'age-corrected' measure of ERα expression by using the dividing line (as defined by Dai *et al*.) between the 'ER/age high' and 'ER/age low' groups (Figure 2a), rather than the zero point, as the baseline. When ranking the genes in the tumor dataset by correlation with this age-corrected ERα, we found a significant enrichment for E2-induced cluster B genes (GSEA nominal *p *= 0.001, FWER *p *= 0.004; Figure 2c) where we had not found such an enrichment when considering ERα independently of age (Figure 2b).

Another way to correct for patient age when relating tumor ERα levels to coordinate expression of E2-inducible genes is to consider a set of tumors for which patients were roughly the same age. From the 226 ERα+ tumor profiles, we considered a subset of 61 profiles from patients with ages 41 to 44. Patients from this age group were evenly divided between the 'ER/age high' and 'ER/age low' (Figure 2a). When ranking the genes in the tumor dataset by correlation with ERα in these patients (without correction for age), we did see an enrichment of cluster B genes within the top ERα correlates by GSEA (nominal *p *= 0.033). Out of the top 720 genes positively correlated with ERα expression (*p *< 0.05), 68 were represented in cluster B (Figure 2e; *p *< 10E-60, one-sided Fisher's exact).

### A significant number of genes induced by estrogen *in vitro *are correlated with PR mRNA expression in ERα+ human breast tumors *in vivo*

**Figure 3:**
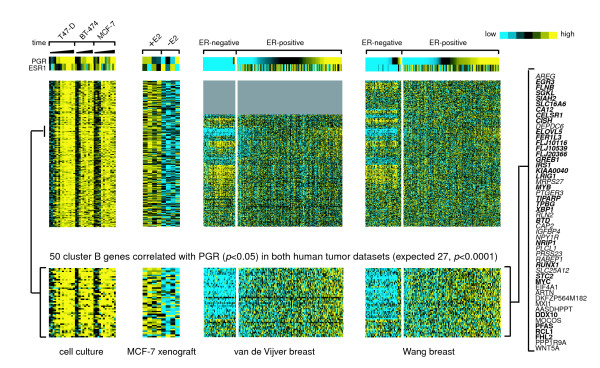
E2-inducible genes are enriched in ER+/PR+ breast tumors compared to ER+/PR- breast tumors. Expression patterns for the cluster B genes (induced by E2 *in vitro *within 4 hours; Figure 1a) are shown alongside the corresponding patterns in MCF-7 xenografts (Figure 1b) and in two independent breast tumor datasets from van de Vijver *et al*. [14] and Wang *et al*. [20]. Genes showing significant correlation with PGR in both van de Vijver and Wang datasets (*p *< 0.05 in each) are highlighted and listed: italics indicate more highly expressed in ER+ compared to ER- tumors (*p *< 0.01); bold type indicates significantly correlated with age-corrected ER expression (*p *< 0.05; see Figure 2). Enrichment *p* value by one-sided Fisher's exact test.

An additional indicator of estrogen signaling activity in breast tumors is the higher expression of known estrogen-inducible targets, such as progesterone receptor (PR). The *PGR *gene encoding PR was among our cluster B genes. PR expression is used clinically in addition to ERα as an important molecular prognostic factor for response to antiestrogen therapy in breast cancer patients, as it indicates the endpoint of estrogen action [19]. In the ERα+ breast tumor profile dataset from van de Vijver *et al*. [14], PGR was correlated with both ERα (Pearson's correlation *p *< 0.01) and age-corrected ERα (*p *= 0.05), and over half of the 674 genes most correlated (*p *< 0.05) with PGR overlapped with the set of 1,338 genes most correlated (*p *< 0.05) with age-corrected ERα (enrichment *p *~ 0). As we anticipated, GSEA showed an enrichment in the top PGR gene correlates for cluster B genes (nominal *p *= 0.001, FWER *p *= 0.004; Figure 2d). We examined a second ERα+ tumor profile dataset from Wang *et al*. [20] and identified 50 genes from cluster B that were significantly correlated with *PGR *(*p *< 0.05) in both the van de Vijver and Wang datasets (Figure 3), where about half the number of genes would have been expected by chance (*p *< 0.0001, Fisher's exact).

### No significant enrichment of E2-regulated genes *in vitro *within genes associated with ERα+ breast tumor status

We next considered ERα-negative tumors along with ERα-positive tumors. One might expect ERα+ tumors to express E2-induced genes more highly compared to ERα- tumors. From the 295 cohort of breast tumor profiles, we selected 79 for which ERα was measured at the protein level, with ERα levels in the ERα+ tumors from 80% to 100% of cells [15]. Surprisingly, when ranking genes in the tumor profile dataset by higher expression in the ERα+ compared to the ERα- tumors, we did not observe any evidence for enrichment of E2-induced genes within the top ERα+ genes (GSEA *p *= 0.405 for cluster B genes; Figure 2d). Out of the top 1,965 expressed genes in ERα+ tumors (*p *< 0.01), 110 were represented in cluster B, which did not represent a significant overlap (*p *= 0.11). We also tried considering a subset of the ERα+ tumors that had the highest PR levels, but could still find no statistical enrichment of E2-regulated genes in the genes distinguishing this ERα+ subset from the ERα- tumors (results not shown). On the other hand, contrary to our initial expectations, we did see an enrichment of E2-induced genes within the top ERα- genes. Out of the top 1,904 expressed genes in ERα- tumors (*p *< 0.01), 132 were represented in cluster B (enrichment *p *= 3.7E-05). If, however, we consider the set of genes that are both induced by E2 in our models and correlated with age-corrected ERα in ERα+ breast tumors, we do find this set to be highly enriched for genes more highly expressed in ERα+ compared to ERα- tumors (Figure 2e).

### Genes induced by estrogen *in vitro *are enriched for transcriptional targets of Myc

**Figure 4:**
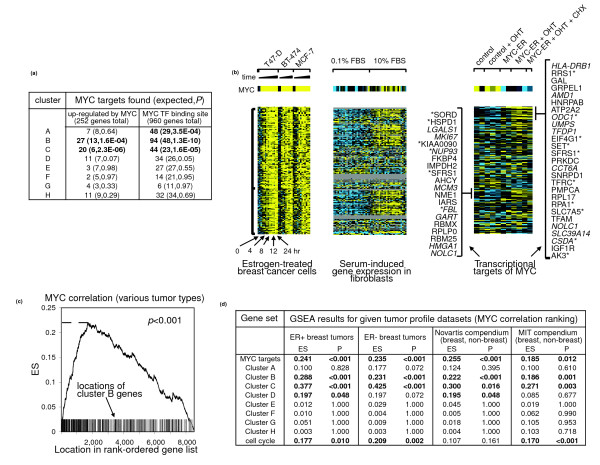
Genes induced by E2 *in vitro *are enriched for transcriptional targets of Myc. A conditional Myc-estrogen receptor (Myc-ER) fusion protein was used to induce Myc transcriptional activity. Conditional activation of Myc occurs upon stimulation with the anti-estrogen 4-hydroxytamoxifen (OHT). Gene expression profiles were taken of primary human fibroblasts with Myc-ER to identify transcriptional targets of Myc [22] (CHX, cycloheximide). **(a) **Enrichment of Myc targets (*p *< 0.01) within each of the distinct clusters of estrogen-regulated genes (Figure 1). Values indicating significance of overlap (*p *< 0.05) between two given genes sets are in bold. **(b) **Expression data matrix for cluster B and C genes that were also represented in the Myc-ER dataset, alongside the corresponding values in both the Myc-ER dataset and in a profile dataset from [26] of human fibroblasts grown in high and low serum conditions, in which Myc is up-regulated in the high serum group. Myc targets (*p *< 0.01) are listed (asterisk indicates has predicted Myc TF site in promoter region; italics indicate cell cycle gene from [13]). **(c) **GSEA of the cluster B genes against the overall ranking of genes according to similarity with *myc *mRNA expression in a compendium of tumor profiles from breast and 10 other tissues types from [24]. **(d) **GSEA results for estrogen-regulated gene clusters A to H, genes induced by Myc-ER+OHT in fibroblasts (*p *< 0.01), and cell cycle genes [13], against gene rankings by Myc correlation in four different datasets (clusters A to H, FWER *p*, Myc and cell cycle, nominal *p*).

We may expect that many of the genes in our E2-regulated gene clusters are not direct transcriptional targets of E2, but rather secondary targets through one or more intermediates. Within the cluster B genes (Figure 1a), with sustained induction by E2 at early time points, about 20% of the 639 genes had the GO annotation of 'nucleic acid binding,' suggesting that many of the genes regulated by E2 encode for transcription factors that may themselves go on to regulate additional genes. One transcription in cluster B is the c-Myc oncogene (*myc*), which is a well-known direct target of estrogen. Knockdown of *myc *expression inhibits estrogen-stimulated breast cancer cell proliferation [21]. It is, therefore, conceivable that a number of genes in our E2-regulated gene clusters would be direct targets of *myc *as well as secondary targets of E2. We referred to two public datasets of putative *myc *target genes (Figure 4a): one mRNA profile dataset of changes caused by activation of *myc *in primary human fibroblasts [22] (252 up-regulated, 238 down-regulated by *myc *with *p *< 0.01), and another dataset of 960 genes with predicted *myc *binding sites in their promoter regions [23]. Both the cluster B and cluster C genes (induced by E2 by 4 hours or 8 hours, respectively) showed high enrichment for both *myc*-induced mRNAs (B, 27 genes, *p *= 1.6E-04; C, 20, *p *= 2.3E-06) and genes with predicted *myc *binding sites (B, 94, *p *= 1.3E-10; C, 44, *p *= 1.6E-05). No enrichment was observed for mRNAs down-regulated by *myc *within mRNAs repressed by E2.

We examined the expression patterns of the E2-regulated gene clusters with respect to *myc *in human tumors. We had found that many of the E2-induced genes were correlated with (age-corrected) ERα mRNA expression in tumors (see above), and so we reasoned that other genes within the estrogen mRNA signature that were specifically involved in the *myc *pathway would be correlated with *myc *in tumors. We examined four mRNA profile datasets of human tumors: the dataset of ERα+ breast tumor profiles used above (Figure 2); a dataset of 69 ERα- breast tumors [14]; a compendium of 174 tumors from 11 different histological types (including breast; the 'Novartis' dataset) [24]; and a second compendium of 138 tumors from 13 different types (the 'MIT' dataset) [25]. In all four datasets, GSEA showed very high enrichment for both cluster B and cluster C E2-induced genes *in vitro *within the top *myc *correlates *in vivo *(Figures 3d and 4d). The *in vitro *set of *myc *targets from [22] (*p *< 0.01) were also significantly correlated with *myc *expression in each of the tumor datasets. In the tumor datasets, *myc *expression was not significantly correlated with ERα mRNA, and the E2-induced genes most correlated with ERα mRNA expression in ERα+ breast tumors were distinct from the E2-induced genes most correlated with *myc *expression (Figures 2e and 3b).

We also considered an mRNA profile dataset measuring the response to serum exposure in human fibroblasts. Gene expression profiles taken from fibroblast cultures derived from 10 anatomic sites were cultured asynchronously in 10% fetal bovine serum (FBS) or in media containing only 0.1% FBS [26]. Many similarities would be expected between the gene expression patterns in fibroblasts in high serum relative to low serum conditions and the expression patterns in ERα+ breast cells in estrogen-rich relative to estrogen-deprived conditions. We observed that fibroblasts in high serum over-expressed *myc *mRNA (*p *= 0.00015), and so we might expect that many of the genes up-regulated in fibroblasts in response to serum would be *myc *targets or closely related to the *myc *pathway. Out of the (unique) 908 E2-induced genes in clusters B and C (Figure 1a), 425 were also significantly up-regulated (*p *< 0.05) in fibroblasts in high serum. Within the E2-induced gene signature, the fibroblast serum-induced genes overlapped with the *myc *target genes (Figure 4b). Conversely, we identified 308 genes in clusters B and C that showed no significant serum induction in fibroblasts (*p *> 0.20); these genes may be more unique to the estrogen signaling pathway rather than general to processes of growth and proliferation.

One possibility to consider is that the associations found here between the estrogen pathway and the *myc *pathway are related to processes of cell division and would be evident in any scenario of highly proliferating cells. However, when examining two public mRNA profile datasets of LNCaP prostate cancer cells stimulated to profilerate with androgen treatment [27,28], *myc *was not found to be up-regulated (Chen dataset *p *= 0.53), and there was no enrichment for *myc *targets within genes inducted by R1881 in LNCaP (Chen dataset enrichment *p *= 0.10). We examined the expression patterns of cell cycle genes from [13] with respect to *myc *expression in tumors; in three of four tumor datasets we did see an enrichment of cell cycle genes within the top *myc *correlates (Figure 4d), though only a few of these were found in the overlap between E2-induced genes and *myc *target genes.

## Discussion

Numerous studies have sought to characterize the transcriptional network associated with the estrogen response using cell culture experiments. However, when relying exclusively on results from *in vitro *studies, concerns may arise that many of the effects observed may be artifacts of the cell line model. Data from *in vitro *models represents dynamic information, where cells can be readily manipulated and the effects observed. Cells can be manipulated to a certain extent *in vivo*, for instance using the tumor xenograft model, though with considerably greater effort. An abundance of gene expression data from human tumors is available to the public domain. However, this type of *in vivo *data represents more static information, where the associations observed may be pathologically relevant, but where cause-and-effect associations cannot be distinguished from mere correlation. An emerging area of DNA microarray analysis of cancer is that of relating profile data from *in vitro *bench experiments to profile data from *in vivo *experimental models and from human tumors. Recent attempts at correlating gene profiles observed in cell line studies, in which oncogenes such as cyclin D1 or k-ras were manipulated, with profiles obtained from patient tumors of various types, have found significant similarities between the *in vitro *and *in vivo *systems [16,17]. These similarities may be subtle in some cases but can be observed using more advanced analytical techniques such as GSEA.

Using global mRNA expression profiling, we have identified transcription networks of hundreds of genes that are either stimulated or inhibited by E2 *in vitro*. However, cultured cells in an *in vitro *environment may differ considerably in behavior compared to those of the same cancer cells that proliferate and form tumors *in vivo *[29]. To determine the physiological relevance of these genes, we compared them with genes showing E2-regulation *in vivo *in xenograft tumors, observing a highly significant number of the same genes being regulated by E2 *in vivo *as well as *in vitro*. Our findings of good agreement between the *in vivo *and *in vitro *estrogen-regulated programs differ from the conclusions reached by one recent study by Harvell *et al*. [30], in which mRNA profiles were taken of ERα+ T47-D-Y human breast cells grown as xenografts in nude mice under the following conditions: E2 supplementation for 8 weeks, no E2 for 8 weeks, and E2 for 7 weeks followed by E2 withdrawal for one week. The Harvell study found little overlap between E2-regulated genes *in vivo *versus *in vitro*. One way to perhaps explain this disparity is the differences in xenograft models and experimental design between the two studies. We suspect that the Harvell study may have selected for genes involved with the adaptation of breast cells to long term estrogen withdrawal, whereas our model focuses on short-term changes in estrogen signaling. We found little overlap between *in vivo *E2-regulated genes in our study and the Harvell study. Out of 1,073 genes induced *in vivo *in our data (*p *< 0.01), only 15 were in the top set of 188 reported by Harvell (9 class I and 6 class II genes, as described in [30]). Furthermore, genes such as *GREB1*, which was previously confirmed by our group by real-timePCR as E2-regulated *in vivo *[5], did not appear to be significant in the Harvell data. These discrepancies may be due to differences in the sensitivity and specificity of the separate DNA microarray platforms used.

We might expect the cluster A and E genes from our *in vitro *data (Figure 1a) to have less agreement with the xenograft data compared to the other clusters, as the A and E genes did not show sustained E2 regulation at 24 hours. Other disparities between our xenograft and *in vitro *data might similarly be attributed to differences in the two experimental models. Since estrogen must be provided for tumor formation, estrogen was withdrawn for 24 and 48 hours in order to generate the non-estrogen stimulated tumor sample. Thus the dynamics of the drop in estrogen concentration over time, the half life of the estrogen induced mRNAs and the changes in gene expression associated with the loss of the proliferative drive will all have contributed to the disparity seen. The expression pattern in the xenograft model is also complicated by the effects of estrogen induced paracrine factors acting on the tumor cells. Currently, we are carrying out experiments to see whether the genes regulated by E2 *in vivo *in both T47D and BT-474 are similar to those showing E2 regulation in MCF-7 xenografts. Based on the above results, we would expect this to be the case, as the *in vitro *E2-stimulated profiles show a remarkable amount of similarity among the three different cell lines.

To determine the clinical relevance of genes regulated by E2 in our experimental models, we compared them with genes correlated with PR expression in human ERα+ breast tumors, with genes correlated with ERα expression, with genes correlated with patient age, and with genes correlated with ERα expression corrected for patient age (which overlap highly with the gene most correlated with PR expression). We demonstrate how early inducible genes of estrogen as a group are expressed together in ERα+ breast tumor with relatively high levels of PR and of ERα for the patient's age, as well as in ERα+ tumors from younger patients compared to older patients. These findings indicate that ERα+ breast cancers with higher levels of estrogen signaling, either through increased ERα expression or the increased estrogen production expected in younger patients, express genes observed to be induced by estrogen *in vitro*. We feel that our results provide significant validation of a widely used *in vitro *model of estrogen signaling as being pathologically relevant to breast cancers *in vivo*. Furthermore, our analysis demonstrates how molecular data can be combined with clinical data such as patient age to uncover associations that may not have been evident when considering either clinical or molecular data alone.

While we observed coordinate expression of E2-regulated genes in ERα+ breast tumors, we did not observe enrichment for E2-induced genes in ERα+ breast tumors compared to ERα- tumors as we might have expected. These observations are consistent with those of previous microarray analyses [10,31], indicating that there may be more to the molecular differences between ERα+ and ERα- tumors than simply a loss of estrogen signaling in the ERα- tumors. This disparity may be accounted for in part by the fact that ERα- tumors are more aggressive and express cell proliferation genes more than ERα-positive tumors [15,32], and, therefore, similarities between cells proliferating in response to E2 *in vitro *and cells within tumors with a high proliferative index would be expected.

We also observed that E2-inducible *myc *expression plays an important role in the estrogen transcription network. Genes induced by E2 were enriched for transcriptional targets of *myc*. The public Oncomine database of cancer gene expression [33,34] showed Myc to be over-expressed in ERα- tumors compared to ERα+ tumors in multiple breast tumor datasets. We find in this study that genes involved in the *myc *network were coordinately expressed with Myc in both ERα- and ERα+ tumors. This observation indicates that the *myc *pathway, which is essential for E2-mediated growth, remains active in the ERα- tumors, and is not shut down along with other components of the estrogen pathway with the loss of ERα expression. The presence of a gene expression signature for *myc *activation within the E2-induction signature may further explain in part the discordance we observed between the E2 signature and the gene expression differences between ERα+ and ERα- breast tumors, as the *myc *portion of the E2 signature is enriched in the ERα- rather than the ERα+ tumors. However, when we removed candidate *myc *targets from the E2 signature, we still did not observe enrichment of the remaining E2-inducible genes within the ERα+ tumors (results not shown). As more experimental *in vitro *signatures become available (for example, signatures of activation for genes other than *myc*), it may be possible to further dissect the E2 signature into meaningful subgroups.

In future studies, we plan to examine the functional roles of specific genes arising from our E2-regulated gene signatures, as well as their relevance to such clinical variables as patient response to therapy. By combining *in vitro *gene expression data on E2 stimulation with our xenograft data, as well as with data from human tumors, we can better select for genes having critical roles in the estrogen response in breast cancer, such as *GREB1*, which has recently been shown by our group to be a critical regulator of hormone dependent breast cancer [5]. We also plan to further explore the gene expression differences between ERα+ and ERα- tumors, and whether genes other than the ER gene might be involved in the ERα- phenotype.

## Materials and methods

### Cell lines, culture conditions and growth assays

Tumor cell lines were maintained and growth assays performed as described previously [5]. For defined estrogen culture experiments, cells were washed and grown in steroid depleted media (phenol red-free IMEM (Improved Minimal Essential Media) supplemented with 10% charcoal stripped calf bovine serum; Valley Biomedical Products, Winchester, VA, USA) as described previously [5].

### MCF-7 xenograft model

MCF-7 cells (about 10^7^) were inoculated subcutaneously in the mammary fat pad region of 6-week old ovariectomized athymic nude mice containing sustained-released E2 pellets (0.72 mg/pellet, 60-day release; Innovative Research of America, Sarasota, FL, USA) as described previously [5]. After 4 weeks, the mice were randomized into control (continued E2 supplementation) or E2 withdrawal (surgical removal of pellet). Tumors were excised 24 and 48 h later, RNA extracted and global gene expression patterns were obtained using the Affymetrix GeneChip as described below. All animal experiments were performed according to a protocol approved by the University of Michigan Guideline for Use and Care of Animals in compliance with US and UKCCCR (Unite Kingdom Coordinating Committee on Cancer Research) guidelines [35].

### RNA extraction

Total RNA was isolated using TRIzol Reagent (Invitrogen, Carlsbad, CA, USA) according to the manufacture's instructions. Yield and quality were determined by spectrophotometry (Beckman DU 640, Beckman Coulter, Inc., Fullerton, CA, USA) and using a Bioanalyzer RNA 6000 Nano chip (Agilent Technologies, Palo Alto, CA, USA). All samples were stored at -80°C. RNA for microarray analysis was labeled and hybridized according to the Affymetrix protocol (Affymetrix GeneChip Expression Analysis Technical Manual, Rev. 3) by the University of Michigan Comprehensive Cancer Center Affymetrix and cDNA Microarray Core Facility.

### Affymetrix microarray analysis

Gene expression patterns were determined using Affymetrix Genechip Arrays. Arrays were normalized and compared using DNA-Chip Analyzer software (dChip) [36]. Array data has been deposited in the public Gene Expression Omnibus (GEO) database, accession GSE3834. The MCF-7 *in vitro *profiles were generated using the Affymetrix U133A platform, while the other profiles were generated on the U133 Plus 2.0 platform; we therefore considered only the 22,283 probe sets shared between the two platforms in our analysis. Gene expression values were log-transformed. Expression values within each cell line were first transformed to standard deviations from the mean. Two-sample *t* tests were performed as criteria for determining significant differences in mean gene mRNA levels between groups of samples. Expression values were visualized as color maps using the Cluster and Java TreeView software [37]. GO annotation terms were obtained and searched for enrichment within gene sets as described in [29], with GO terms applying to 10 or more genes being considered for enrichment, and with a '*q*-value,' using the method in [38], indicating significance after correcting for multiple term testing. Significance of overlap between two independently derived gene sets (for example, a given estrogen-regulated gene cluster and Myc target genes) was determined by one-sided Fisher's exact test.

When mapping genes across our estrogen mRNA profiling datasets (time course, xenograft, and datasets from [5]), the Affymetrix probe Id (corresponding to a specific mRNA transcript) was used. When mapping genes from our estrogen datasets to independent datasets from other laboratories and other platforms, the gene symbol was used; where a gene was represented multiple times on a given platform, the gene with the most significant patterns of interest was used (for example, most correlated with ERα expression in either direction for the dataset from [14]). For the Myc profiling dataset from [22], values within each of the three experiments were first transformed to standard deviations from the mean, and a *t* test determined significance between a conditional Myc-ER fusion protein activated by 4-hydroxytamoxifen (Myc-ER+OHT) and control+OHT groups. The xenograft profiling experiments were carried out on two separate dates (two profiles +E2, two profiles -E2 on each occasion), and so expression values within each batch of profiles were first transformed to standard deviations from the mean before comparing the +E2 and -E2 groups.

GSEA was carried out essentially as described in [16], with the exception that our tests were one-sided (as in [17]). Briefly, the common population of genes represented in the given *in vivo *tumor profile dataset and our E2 Affymetrix dataset were ranked based on a particular metric applied to the expression values in the tumor dataset (for example, correlation with ERα expression). The significance of the positions of a given gene set of interest (for example, estrogen-regulated gene cluster) being located near the top of the ranked list was assessed by the Kolmogorov-Smirnov score, from which an 'ES' statistic was derived. If a gene was represented more than once on a given platform, then the highest ranking for the gene was used for the GSEA. The significance of the ES statistic was determined by carrying out the same analysis using 1,000 random permutations of the sample labels in the tumor dataset.

## Additional data files

The following additional data are available with the online version of this paper. Additional data file 1 is a table providing a complete list of genes regulated by E2 *in vitro *in each of the eight gene clusters shown in Figure 1.

## Supplementary Material

Additional File 1 Complete list of genes regulated by E2 *in vitro *in each of the eight gene clusters shown in Figure 1. Click here for file
